# Correction: Saba et al. Anti-Melanogenic Effects of Korean Red Ginseng Oil in an Ultraviolet B-Induced Hairless Mouse Model. *Molecules* 2020, *25*, 4755

**DOI:** 10.3390/molecules30061229

**Published:** 2025-03-10

**Authors:** Evelyn Saba, Seung-Hyung Kim, Yuan Yee Lee, Hyun-Kyoung Kim, Seong-Soo Roh, Yi-Seong Kwak, Chae-Kyu Park, Sung-Dae Kim, Man Hee Rhee

**Affiliations:** 1 Department of Veterinary Medicine, College of Veterinary Medicine, Kyungpook National University, Daegu 41566, Republic of Korea; 2 Institute of Traditional Medicine and Bioscience, Daejeon University, Daejeon 34520, Republic of Korea; 3 Department of Food Science and Engineering, Seowon University, Chungbuk 28674, Republic of Korea; 4 College of Korean Medicine, Daegu Haany University, Daegu 42158, Republic of Korea; 5 R&D Headquarters, Korean Ginseng Cooperation, Daejeon 34520, Republic of Korea; 6 Research Center, Dongnam Institute of Radiological and Medical Sciences, Busan 46033, Republic of Korea

## Error in Figure

In the original publication [1], there was a mistake in Figure 7C as published. The M-T staining pictures of KRG-O 1% and 0.5% were placed in the opposite positions. The corrected Figure 7 appears below.

## Conflicts of Interest

In the original publication [1], the Conflicts of Interest statements of Yi-Seong Kwak and Chae-Kyu Park were not included. The updated Conflicts of Interest should read as follows: Authors Yi-Seong Kwak and Chae-Kyu Park were employed by the company Korean Ginseng Cooperation. The remaining authors declare that the research was conducted in the absence of any commercial or financial relationships that could be construed as a potential conflict of interest. 

The authors state that the scientific conclusions are unaffected. This correction was approved by the Academic Editor. The original publication has also been updated.

## Figures and Tables

**Figure 7:**
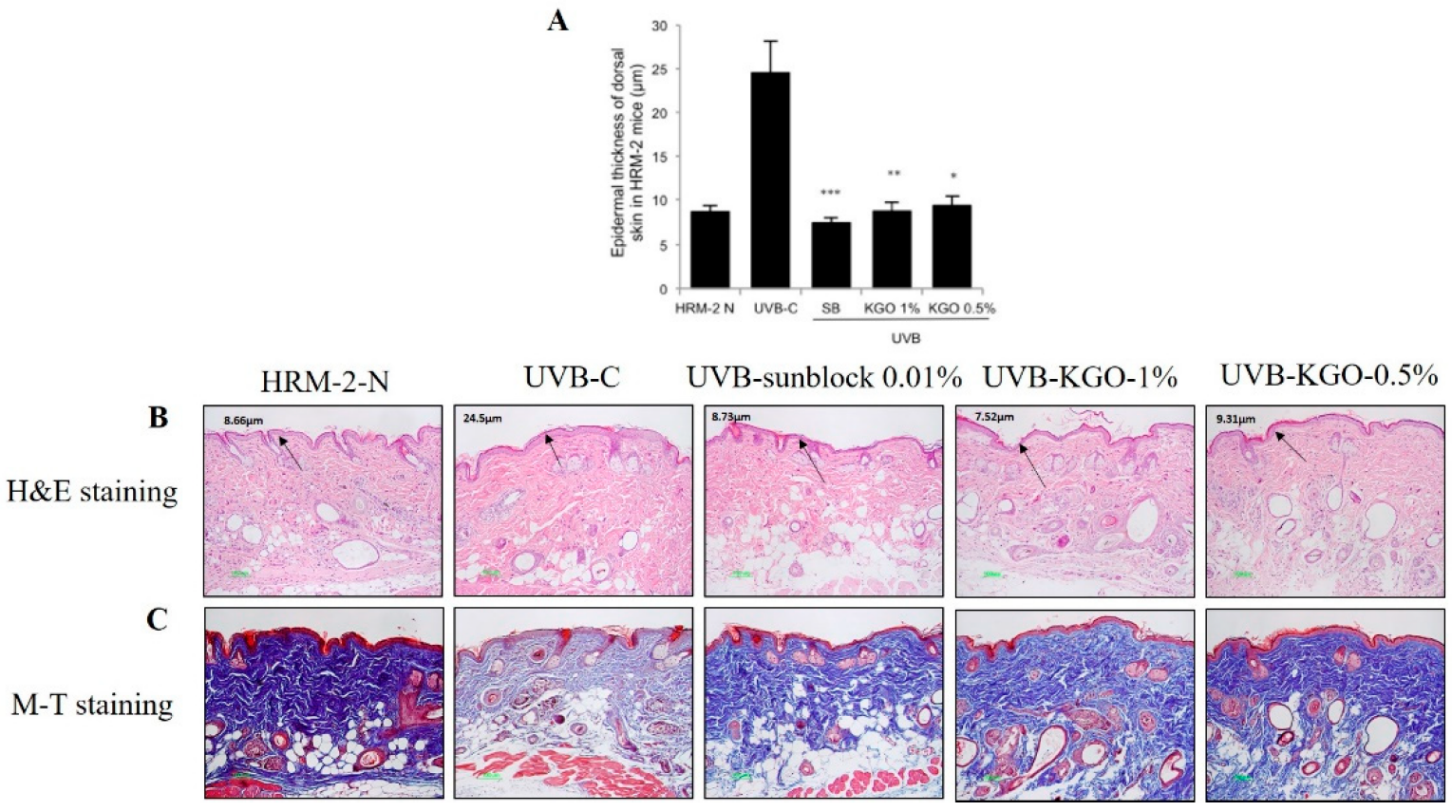
Effects of KGO ointment on epithelial thickness and changes in collagen fibre in HRM-2 mice. After 5 weeks, the epithelial thickness of the skin was observed after staining with H&E (**A**,**B**). A significant reduction was found in the epidermal thickness of the KGO ointment-treated groups. Values in the bar graphs are ± SEM from three independent experiments. *** *p* < 0.001, ** *p* < 0.05, and * *p* < 0.01 when compared with the UVB control. (**C**) The intensity of M-T staining was decreased in the UVB-control group compared to the normal group, suggesting that collagen fibre degradation progressed, and wrinkle formation accelerated. However, the amount of collagen fibres in the KGO ointments (1 and 0.5%) and positive control group increased, indicating that KGO ointments reduced the amount of collagen degradation. The normal mice are abbreviated as HRM-2 N; the UVB control is abbreviated as UVB-C; sunblock is abbreviated as SB.
